# Predicting the current and future distributions of *Pennisetum alopecuroides* (L.) in China under climate change based on the MaxEnt model

**DOI:** 10.1371/journal.pone.0281254

**Published:** 2023-04-04

**Authors:** Yuandong Xu, Ruifen Zhu, Lifang Gao, Dejun Huang, Yan Fan, Chang Liu, Jishan Chen

**Affiliations:** Institute of Pratacultural Science, Chongqing Academy of Animal Sciences, Rongchang, China; Amity University, INDIA

## Abstract

*Pennisetum alopecuroides* (L.), one of the important exotic plants, gives great economic value to animal husbandry in China. In order to study the distribution of *Pennisetum alopecuroides* (L.) in China and its response to climate change, based on the distribution records of *Pennisetum alopecuroides* (L.), our study used the Maximum Entropy (MaxEnt) model and geographic information system (GIS) methods, combined with environmental factors such as climate and terrain, to predict the potential distribution areas suitable for *Pennisetum alopecuroides* (L.) under current and future climate scenarios. The results showed that annual precipitation was the most important factor affecting the distribution of *Pennisetum alopecuroides* (L.). In current climate scenario, the total area of suitable for *Pennisetum alopecuroides* (L.) growth was about 576.5 km^2^, accounting for about 60.5% of the total land area of China. Among all the suitable areas, the area of low, middle and high fitness areas accounted for 5.69%, 20.55% and 33.81% of the total area respectively. In future climate scenarios (RCP4.5), the suitable area of *Pennisetum alopecuroides* (L.) would decrease with climate change, showing a clear trend of northward expansion in China. A concentrated and contiguous distribution region for *Pennisetum alopecuroides* (L.) would appear in northeast China. The model was tested by the receiver operating characteristic curve (ROC), and the average area under the curve of ROC of the training set was 0.985, which was reliable. This work provided an important reference and theoretical basis for the efficient utilization and plant regionalization of *Pennisetum alopecuroides* (L.) in future.

## 1. Introduction

Climate is one of the most important ecological factors that determine the distribution of plants [1], and the response of vegetation to climate change and the regional change of plant distribution under the influence of climate are the hot issues of biogeography and macroecology [2]. The global average surface temperature has climbed by 1°C since the latter half of the 19 century, and the increase of it is supposed to reach, or even exceed 1.5°C between 2021 and 2040. Climate warming will greatly affect the species distribution and ecosystem functions of natural ecosystems in China [3]. Therefore, the research on the prediction of species distribution under the background of climate change can provide strategic basis for agricultural production, biodiversity conservation and ecosystem sustainable development.

Advances in Geographic Information System (GIS) technology and related statistical modeling provide new tools for biogeography [4]. Species distribution models (SDM) are commonly used to predict the potential biogeographic extent of climate change and its consequences [5–7]. SDM (SDM) is a kind of quantitative model based on niche theory to study the environmental tolerance of species on the basis of known distribution points and related environmental factors [8, 9]. Combined with global climate models (GCM), SDM can predict the range change of species distribution under future climate scenarios. Commonly used SDM includes bioclimate analysis and prediction system (BIOCLIMM), genetic algorithm for rule-set production (GARP) and maximum entropy modeling (MaxEnt). Among them, MaxEnt model still has the advantage of good prediction when the distribution of species is small or the number of species is uncertain and the correlation with environmental variables is unknown [10]. For example, Zhang et al. [11] found that the MaxEnt model can accurately predict the distribution of Populus Euphratica, the main environmental factors are temperature and precipitation factors. Yan et al. [12] compared the prediction effect of MaxEnt and GARP model on the potential risk area of plague, which shows that MaxEnt is more accurate and GARP model has a wider range of prediction.

*Pennisetum alopecuroides* (L.) is the fast-growing plant species in tropical and subtropical area with high forage value and wide distribution [13, 14]. *Pennisetum alopecuroides* (L.) is considered as one of the major alien species in the south area and has the ability of wide distribution. For example, according to the recent investigations of forage resources in Sichuan China, the planting area in Sichuan is the second place, accounting for 28.9% of the total plantation area. Generally speaking, the research on the distribution of *Pennisetum alopecuroides* (L.) mainly focuses on the features of the biased toward the south. For example, Wang et al. [15] noted that *Pennisetum alopecuroides* (L.) is highly asexual and has a wide range of adaptations. However, under the influence of global warming, it is still uncertain how to predict the suitable area according to the geographical distribution characteristics of *Pennisetum alopecuroides* (L.) in China.

In this paper, the distribution pattern of *Pennisetum alopecuroides* (L.) in China and the influence of climate change on it are analyzed on the macro-scale. This paper mainly solves two scientific problems: 1) the distribution of potential suitable areas of different grades of *Pennisetum alopecuroides* (L.) in the current period; 2) the prediction of the change trend of the range of suitable areas of *Pennisetum alopecuroides* (L.) in the future climate scenarios. This is of great significance to the protection and artificial cultivation of Chinese forage, the efficient utilization of Chinese forage resources and the selection of planting regionalization.

## 2. Material and methods

### 2.1. Presence records of *Pennisetum alopecuroides* (L.)

**Fig 1:**
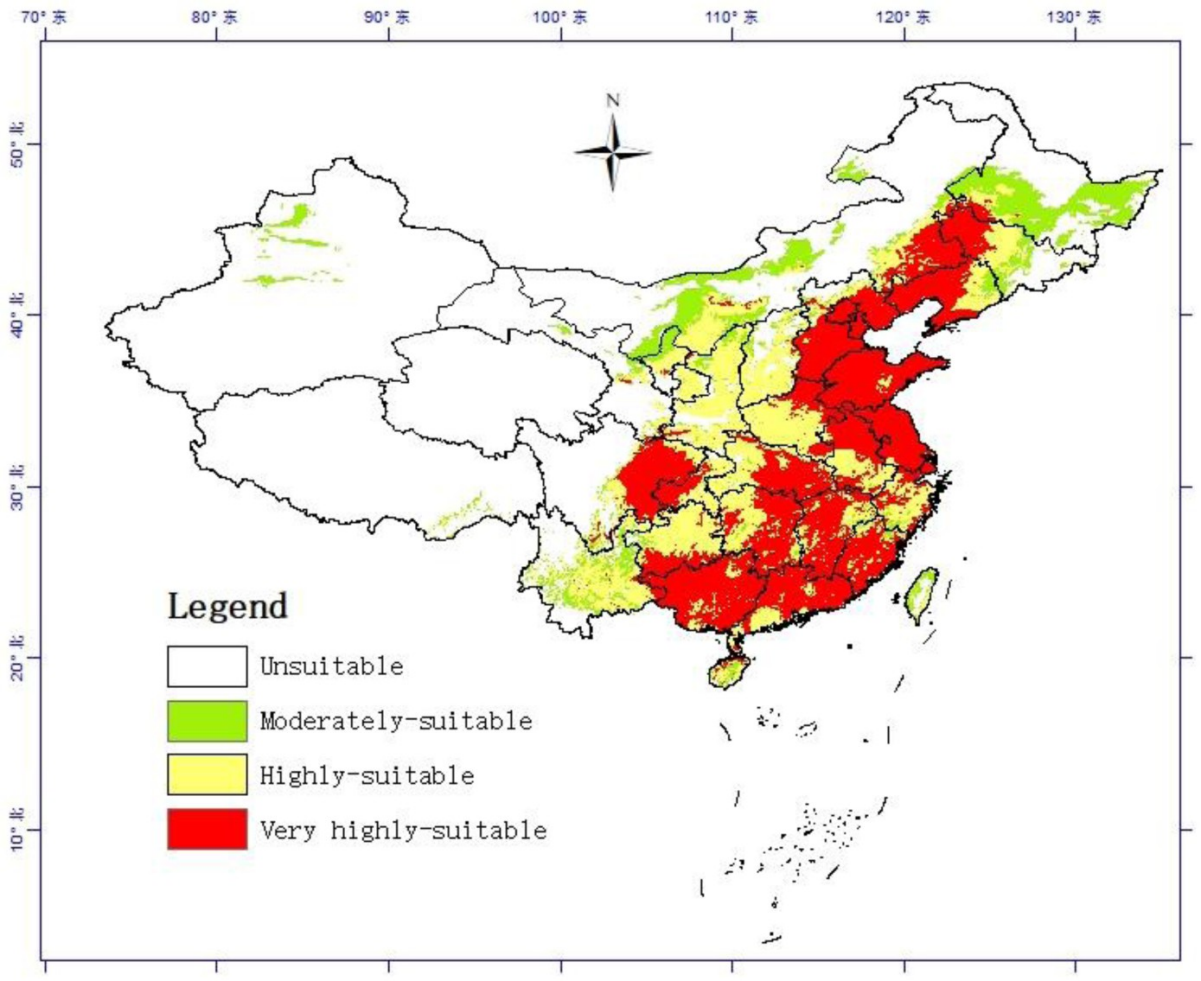
Potentially suitable climatic distribution of *Pennisetum alopecuroides* (L.) under current climate condition in China. The probability of *Pennisetum alopecuroides* (L.) is shown by the color scale in the area. Red indicates a very high suitable area with a probability of higher than 0.56, yellow indicates a highly suitable area with a probability of 0.35–0.56, green indicates a low suitable area with a probability ranging from 0.16 to 0.35, and white represents unsuitable areas. (For interpretation of the references to color in this figure legend, the reader is referred to the Web version of this article.).

The construction of niche model needs sufficient existing presence records of species [16]. In order to obtain the record of *Pennisetum alopecuroides* (L.), most of the sampling sites were established by field investigations using a GPS [17]. The presence records were transformed to uniform latitude and longitude coordinates [18]. At the same time, the distribution data of *Pennisetum alopecuroides* (L.) mainly come from the Global Biodiversity Information Facility (GBIF, http://www.gbif.org/), Chinese Virtual Herbarium (CVH) and literature search. In order to make the sample data more representative of the current period, this study only selected the sample data after 1970. For the distribution data that does not have latitude and longitude but is collected to the village or more fine level, the coordinates of the collection place are queried by Google Earth as distribution points, and the repeated distribution data of latitude and longitude are filtered manually. In order to prevent the sampling error from leading to over-density of local distribution points and over-fitting of the model, many distribution points which are too close to each other are eliminated in ArcGIS by setting buffer zone and cross analysis to ensure that each 2.5 Min 2.5 min grid has at most one distribution point. Finally, a total of 167 records of *Pennisetum alopecuroides* (L.) distribution in China (Fig 1) was used to establish the prediction model (S1 Appendix) based on the software of MaxEnt [19, 20].

### 2.2. Environmental variables

It was important that environmental variables for habitat and species niche distributions [21, 22]. The 19 bioclimatic variables (1970~2000) (Table 1) used in this work were downloaded from Worldclim (www.worldclim.org) with a spatial resolution of approximately 2.5 min (4.5 km). Due to the lack of future terrain data under climate change, the study assumes that the national terrain will not change during the forecast period.

**Table 1:** Modeling environment variables for potential geographic distribution of *Pennisetum alopecuroides* (L.).

Code of environmental variables	Description for environmental variables	Unit	Choose or not
Bio1	Annual mean temperature	°C	Yes
Bio2	Mean diurnal range	°C	No
Bio3	Isothermality	-	Yes
Bio4	Temperature seasonality	-	No
Bio5	Max temperature of warmest month	°C	Yes
Bio6	Min Temperature of coldest month	°C	Yes
Bio7	Temperature annual range	°C	No
Bio8	Mean temperature of wettest quarter	°C	Yes
Bio9	Mean temperature of driest quarter	°C	Yes
Bio10	Mean temperature of warmest quarter	°C	Yes
Bio11	Mean temperature of coldest quarter	°C	Yes
Bio12	Annual precipitation	mm	Yes
Bio13	Precipitation of wettest month	mm	Yes
Bio14	Precipitation of driest month	mm	Yes
Bio15	Precipitation seasonality	-	Yes
Bio16	Precipitation of wettest quarter	mm	Yes
Bio17	Precipitation of driest quarter	mm	Yes
Bio18	Precipitation of warmest quarter	mm	Yes
Bio19	Precipitation of coldest quarter	mm	No

The Fifth Assessment Report was released by the Intergovernmental Panel on Climate Change (IPCC) in 2013, and four representative concentration pathways (RCPs) were published in the report. RCP2.6 (the minimum scenario for GHG emissions), RCP4.5 (the intermediate scenario for GHG emissions) and RCP8.5 (the highest scenario for GHG emissions) were used to test the habitat suitability ranges of *Pennisetum alopecuroides* (L.) in the 2050s (2041–2060) and 2070s (2061–2080).

Since there are many significant correlations among the extracted environmental variables, not all variables were required. Therefore, in establishing models, the jackknife test in MaxEnt (version 3.4.1) model was used to evaluate the contribution rate of each variable and the variables with small contributions were eliminated [23]. We used Pearson’s correlations to examine the cross-correlation of the remaining meteorological data. Based on Xu et al.’s method [7], highly correlated variables (≥0.8) were removed to improve the testing accuracy and decrease the phenomenon of over-fitting. Finally, a lot of 15 environmental variables were retained to simulate predictions of the current and future potential suitable areas.

In order to eliminate the strong correlation between some environmental factors that lead to over-fitting of the model, all environmental factors are imported into MaxEnt model to run according to the default parameters, the Spearman correlation analysis was used to analyze the remaining factors. If there are two or more environmental factors whose correlation is greater than 0.8, the factors with higher contribution rate are selected to participate in the model according to the results of the model trial run. Finally, 15 environment variables are retained to participate in the model operation (Table 1).

### 2.3. MaxEnt modeling

The preprocessed data including distribution records of *Pennisetum alopecuroides* (L.) and environment variables are imported into MaxEnt software, 75% of the *Pennisetum alopecuroides* (L.) records were used to create training data and the remaining 25% were used to the test date. The operation was repeated 6 times.

We adjusted the regularization multiplier (RM) and feature combination (FC) parameters by calling the ENMeval package in R software [24]. Currently MaxEnt model provided 5 features: linear (L), quadratic (Q), hinge (H), product (P), and threshold (T). In its default setting, RM = 1, FC = LQH. In order to optimize the MaxEnt model, in our study, RM was set to 0.5~3, increasing by 0.5 each time. Six feature combinations were adopted, including L, LQ, H, LQH, LQHP and LQHPT. Then, the above 36 parameters were combined into ENMeval package for verification.

In the MaxEnt model, receiver operating characteristic curve (ROC) is used to measure the accuracy of the model. The area between the ROC curve and the ABSCISSA axis is called AUC (area under ROC), this value is the most optimal model accuracy test index at present. AUC scores could be divided into five categories, which were 0.50 ~ 0.60 (failing), 0.60 ~ 0.70 (poor), 0.70 ~ 0.80 (fair), 0.80 ~ 0.90 (good), 0.90 ~ 1.00 (excellent) [25].

In this study, a group of single-factor response curves produced by MaxEnt model without considering other environmental variables were used to determine the relationship between the variation of environmental factors and the fitness of *Pennisetum alopecuroides* (L.). The percentage of contribution of environmental factors in the prediction of MaxEnt model is usually used to characterize the importance of a single environmental factor, and the higher the value is, the greater the contribution of the factor to the simulation results Permutation importance comes from the ranking of random variables based on training sites and environmental data. The larger the value, the larger the proportion of environmental variables collected.

We used ArcGIS software to extract the output results of MaxEnt model and analyzed the climatic suitability of *Pennisetum alopecuroides* (L.) in China. Based on the division method of the evaluation probability in the IPCC report [26], the running result of the model is a fitness map with raster layer value range, and the minimum presence (minimum training presence) or the method of 10^th^ percentile is used for thresholding is used in ArcGIS with the re-classification tool, according to the definition of fitness degree from low to high, the results of current and future prediction are divided into: unsuitable (<0.16), moderately suitable (0.16 ~ 0.36), highly suitable (0.36 ~ 0.56) and very high suitable (>0.56). The administrative boundaries of China used in this study were drawn from the standard base map (http://www.ngcc.cn). Maxent software download at http://biodiversityinformatics.amnh.org/open_source/Maxent/. ArcGIS software version is 10.4. SPSS software version is 17.0.

## 3. Results

### 3.1. Model performance and selection of environmental variables

After optimization with ENMeval (FC = LQHPT, RM = 2), AUCDIFF was 0.015, delta AICc was 0, which were significantly lower than the default settings (Table 2). It shows that the optimized model significantly reduces overfitting to the native distribution data. Meanwhile, the prediction accuracy and stability of the optimized model were higher than the default setting, and the prediction accuracy reached the standard of “excellent”, which means that the prediction result of this study had high reliability. These results also confirmed that the MaxEnt model has an excellent prediction effect on the geographical potential distribution areas of *Pennisetum alopecuroides* (L.) in China.

**Table 2:** Model performance under default and optimized settings.

Treatment	Default	Optimization
FC	LQH	LQHPT
RM	1	2
Mean AUC	0.932	0.985
AUC_DIFF_	0.068	0.015
delta AICc	149.121	0

Note: RM regularization multiplier, FC feature combination, AUC area under the ROC curve, AUC_DIFF_ the difference between the training AUC and the test AUC, AIC, Akaike information criterion.

The contributions rates of each environmental variable to the modeling process were evaluated through the jackknife test and the variables with small contributions were eliminated.

Table 3 have shown the contribution and permutation importance of the 15 environmental variables that contributed significantly to the distribution of *Pennisetum alopecuroides (L*.*)*. Precipitation in the warmest quarter (bio18, 30.8%), Isothermality (bio3, 23.4%), Mean temperature of wettest quarter (bio8, 23.2%), Mean temperature of warmest quarter (bio10, 9.8%), Precipitation seasonality (bio15, 3.8%), Mean temperature of driest quarter (bio9, 2.4%), Annual mean temperature (bio1, 2.4%), the other environmental factors contributed less than 2% (Table 3). Therefore, their cumulative contribution rate of the key environmental variables is 95.8%.

**Table 3:** Contribution and ranking importance of the environmental variables.

Code of environmental variables	Contribution (%)	Permutation importance (%)
Bio18	30.8	18.4
Bio3	23.4	24.5
Bio8	23.2	19.6
Bio10	9.8	8.5
Bio15	3.8	13.9
Bio9	2.4	1.7
Bio1	2.4	1.3
Bio5	1.8	0.9
Bio13	1	0.5
Bio16	0.5	0.2
Bio12	0.4	2.5
Bio14	0.2	0.9
Bio6	0.2	0
Bio11	0.1	0.1
Bio17	0.1	0.3

### 3.2. Current potential distribution of *Pennisetum alopecuroides* (L.) in China

Under the current climate, the suitable area of *Pennisetum alopecuroides* (L.) is mainly distributed in the front line of Hu Huanyong and south of China. The suitable area accounts for about 60.05% of the total land area of China. The area of low, middle and high fitness areas accounted for 5.69%, 20.55% and 33.81% of the total area respectively (Table 4).

**Table 4:** Suitable areas of *Pennisetum alopecuroides* (L.) in different periods(×10^4^km^2^).

Time	Total suitable area	Unsuitable area	Low suitable area	Medium suitable area	High suitable area
Current	576.5	383.5	54.6	197.3	324.6
2050s (rcp2.6)	422.6	537.4	60.8	154.3	207.5
2050s (rcp4.5)	416.8	543.2	63.8	150.7	202.3
2070s (rcp2.6)	420.5	539.5	65.7	149.2	205.6
2070s (rcp4.5)	415.2	544.8	66.5	148.7	200.0

In terms of the distribution of provinces, the high suitability areas are concentrated from East to West in the province-level division of Shandong, Jiangsu, Zhejiang, Fujian, Jiangxi, Guangdong, Guangxi Zhuang Autonomous Region, Chongqing, Guizhou, etc., and in Hunan, Hubei and the eastern part of Sichuan, southern Shaanxi, eastern and northeastern Yunnan and parts of the central and southern Anhui Mountain Distribution, in Henan, Hainan and Taiwan and other places scattered distribution. Moderate-growing areas fill in some gaps in the contiguous distribution of high-growing areas, such as Sichuan Basin, Jianghan Plain, etc. The low-adapted areas are mainly distributed around the middle and high-adapted areas from north, west and northeast. The North reaches to the north of Qin Mountains-Huai River and Inner Mongolia. It is worth noting that in the southeast of the Tibet, such as Nyingchi and Sannan, there is a concentrated distribution of suitable areas for *Pennisetum alopecuroides* (L.) (Fig 1). This area belongs to the South Tibet Valley and has a subtropical climate, it is true that there were records of the distribution of *Pennisetum alopecuroides* (L.) in this region.

### 3.3. Future prediction potential distribution of *Pennisetum alopecuroides* (L.) in China

**Fig 2:**
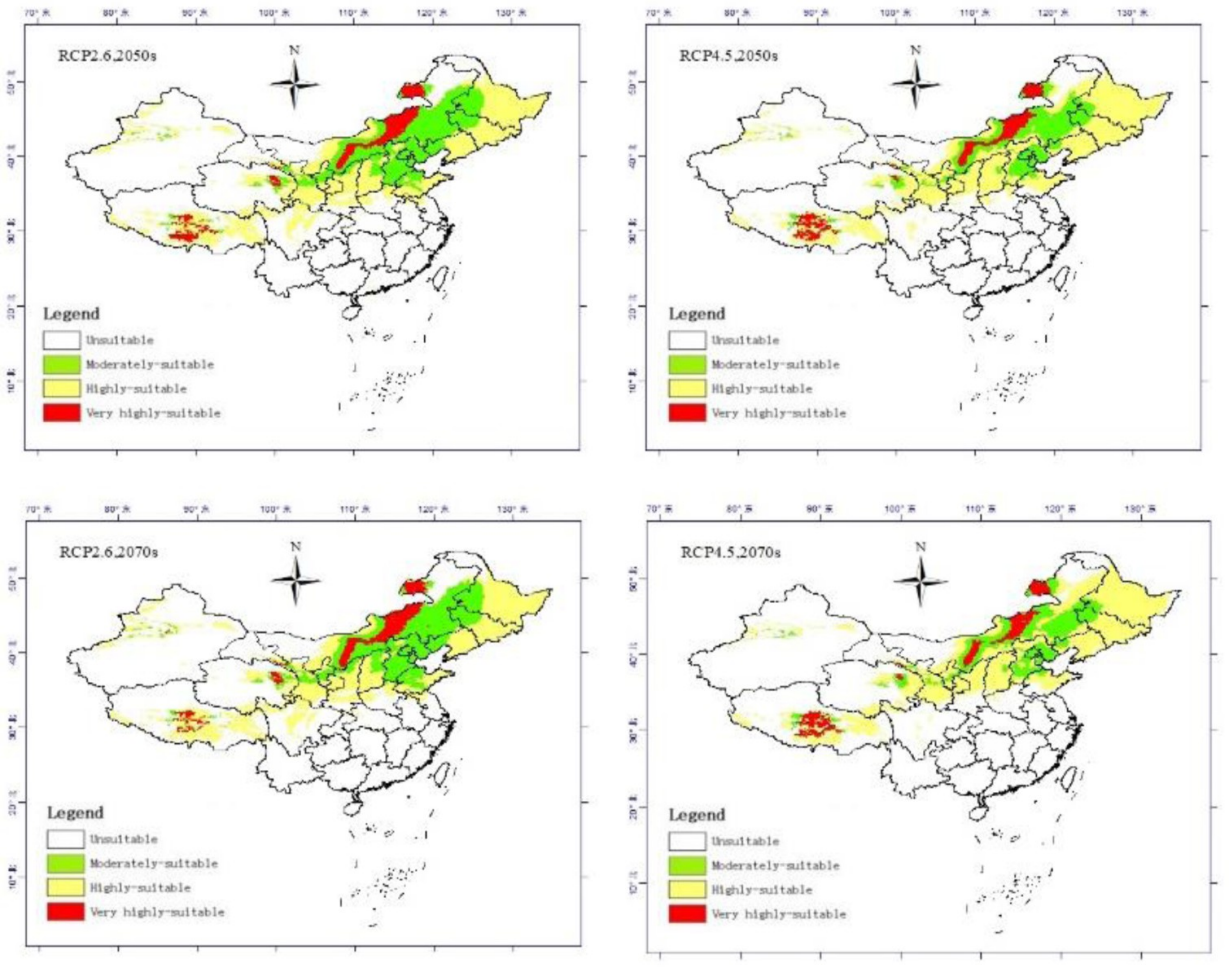
Spatial distribution of suitable areas for different grades of *Pennisetum alopecuroides* (L.) under future climate scenarios.

The predicted future suitable climate distribution of *Pennisetum alopecuroides* (L.) under RCP2.6 (2041–2060) and RCP4.5 (2061–2080) climate change scenarios are shown in Fig 2. In the 2050s and 2070s, there were significant differences between the current and predicted areas. In particular, a large decrease in area was found compared to the current area (Table 4). The very high suitable area for *Pennisetum alopecuroides* (L.) under the RCP2.6 and RCP4.5 scenarios in the mid-century (2050) were projected to decrease by 21.61%, and 21.07%, respectively. The region of the moderately suitable area was decrease by 16.07%, and 15.70%. The region of the low suitable area was decrease by 6.33%, and 6.65%. In the end century (2070), the high suitable area for *Pennisetum alopecuroides* (L.) under the scenarios of RCP2.6, RCP4.5 were projected to increase by 21.42%, and 20.83%, respectively. The region of medium suitable area was increase by 15.54%, and 15.49%. In addition, the low suitable area under the two RCPs were expected to be reduced by 6.84%, and 6.93%.

As can be seen from Fig 2, in the 2050s the southern region including Yunnan, Sichuan Basin, Hainan Island and Taiwan Island, almost all of them turned into unsuitable zones, and the overall fitness zone expanded northward under the RCP2.6 scenario. In the 2070s, this trend has intensified and developed, the suitable area expanded northward to northeast of China, and formed the center of high suitable area in northeast of Inner Mongolia, western Qinghai and Southern Tibet. The adaptation of 2050s under RCP4.5 scenario was more significant shrink from south to north than that under RCP2.6 scenario in suitable zones. In RCP4.5 scenario, 2070s had obvious suitability degradation trend and the suitable area continued to expand northward. On the whole, the suitability of *Pennisetum alopecuroides* (L.) in China will be significantly reduced under the climate change in the future, but the intense climate warming and carbon emission represented by RCP4.5 will cause the suitable area of *Pennisetum alopecuroides* (L.) to shrink from south to north in 2061–2080.

### 3.4. Contribution of environmental variables affecting the geographical distribution of *Pennisetum alopecuroides* (L.)

**Fig 3:**
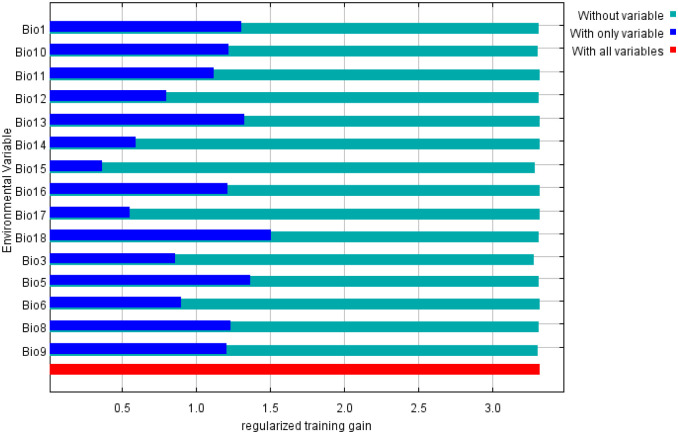
Results of jackknife test of the relative importance of predictor environmental variables in MaxEnt model for *Pennisetum alopecuroides* (L.) in China.

**Fig 4:**
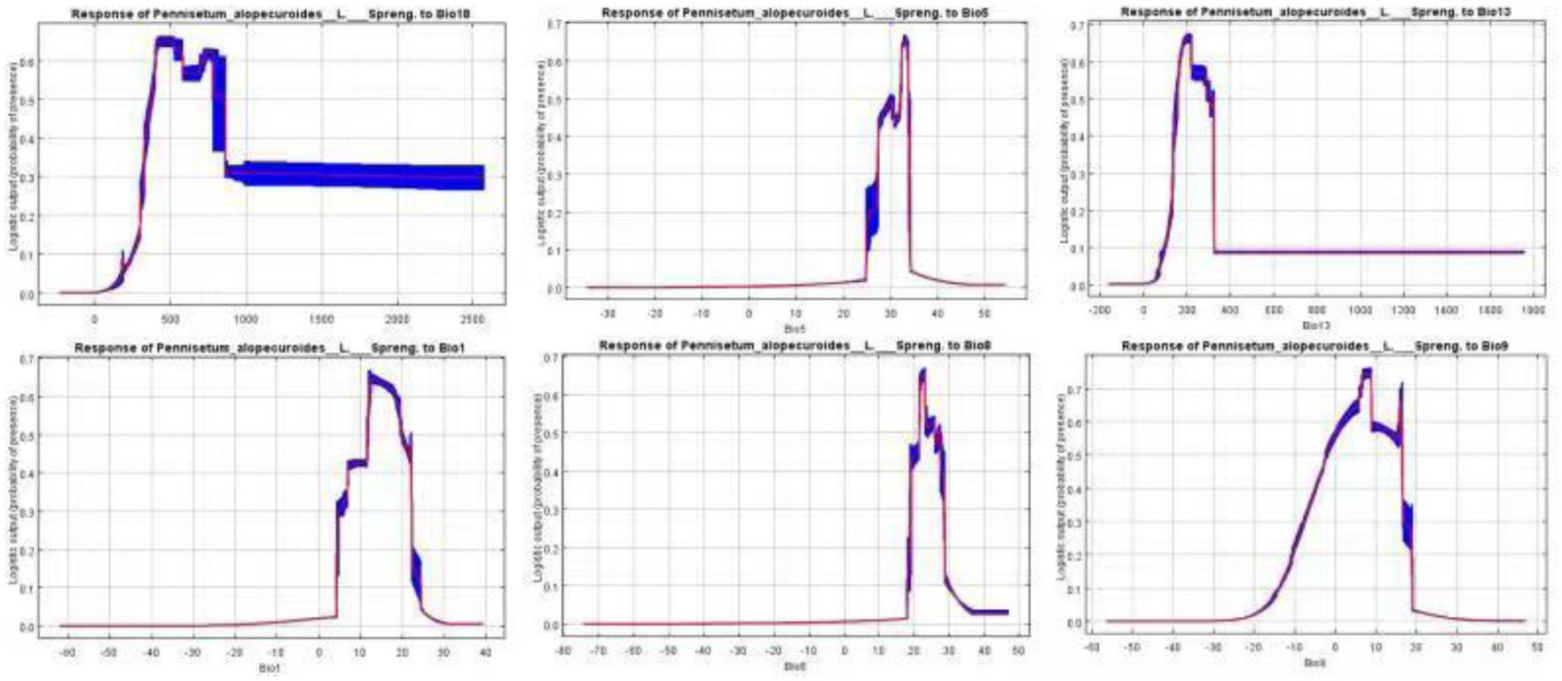
Response curves of climate suitability for the major environmental factors.

Based on the results of the jackknife test, the “with only variable”, “without variable” and “with all variables” cases were showed to reveal the environmental variablesimpacts on *Pennisetum alopecuroides* (L.) suitable distribution area (Fig 3). The environmental variable with the highest gain was bio18 (the precipitation in the warmest quarter), with the regularized training gain of more than 1.5. The max temperature of warmest month (bio5), annual mean temperature (bio1), mean temperature of wettest quarter (bio8) and precipitation of wettest month (bio13) were also important environmental variables, and the regularized training gains were all above 1.2. The environment variables response curve is output by the MaxEnt model. As can be seen from Fig 4, the top 6 environmental variables with the contribution rate from high to low are bio18 (precipitation of warmest quarter), bio5(max temperature of warmest month), bio13(precipitation of wettest month), bio1(annual mean temperature), bio8 (mean temperature of wettest quarter), bio10 (mean temperature of warmest quarter), and bio9 (mean temperature of driest quarter), which were the dominant variable affecting the distribution of *Pennisetum alopecuroides* (L.).

## 4. Discussion

The MaxEnt model is based on the theory of maximum entropy and analyzes the distribution state of species when the entropy reaches a maximum under restricted conditions [27, 28]. In our study, the potential geographical distribution map of *Pennisetum alopecuroides* (L.) in China was obtained for the first time using the MaxEnt model. The model has excellent fitting ability (AUC = 0.985), which indicates that the model can objectively predict the potential suitable growth area of *Pennisetum alopecuroides* (L.) in China. Moreover, this study discarded the highly autocorrelated climate variables, so there was on problem of model overfitting.

The results show that the suitable area of *Pennisetum alopecuroides* (L.) mainly distributes in the subtropical zone under the current climate, and the middle and high suitable area concentrates in the east of Sichuan Basin and south of Qin Mountains-Huai River. The top 7 environmental factors affecting the distribution of *Pennisetum alopecuroides* (L.) from high to low are precipitation of warmest quarter (bio18), max temperature of warmest month (bio5), precipitation of wettest month (bio13), annual mean temperature (bio1), mean temperature of wettest quarter (bio8), mean temperature of warmest quarter (bio10), and mean temperature of driest quarter (bio9). The model predicted that the annual average precipitation from 500 to 800 mm was the highest for *Pennisetum alopecuroides* (L.) (>0.6) (Fig 4), and the contribution rate of the max temperature of the warmest month was the second highest, which indicated that *Pennisetum alopecuroides* (L.) was highly sensitive to max temperature of the warmest month. It is consistent with the results of Zhang et al. [29]. But it is distinguished from the results that *Pennisetum alopecuroides* (L.) is not so sensitve to water stress compared with salt stress [30]. Among the top 7 environmental variables, the fourth to seventh positions were all temperature dependent variables. The response of *Pennisetum alopecuroides* (L.) to the max temperature of the warmest month was the most sensitive, which indicated that the extreme max temperature was probably the main factor limiting the southward expansion of *Pennisetum alopecuroides* (L.). The response curve of environmental variables showed that the lowest temperature of the warmest month above 34.5 °C would lead to the decrease of the adaptability of *Pennisetum alopecuroides* (L.), which could explain the phenomenon of low or even no adaptability of *Pennisetum alopecuroides* (L.) in some high temperature and humid areas.

In the past few decades, although climate warming causes species of plants and animals to migrate to the Poles along the latitudinal gradient, the direction and extent of latitudinal changes in species distribution has been much debated over the past few decades [31–34]. The results showed that the distribution of *Pennisetum alopecuroides* (L.) in south China had the trend of expanding to the west low latitude (near 27° N) in the low scenario (RCP2.6) and the middle scenario (RCP4.5). It has been proved that the species whose geographic distribution is dominated by precipitation and the species whose distribution is dominated by temperature move in different directions, the indexes of water condition and water-heat correlation in climate change have great influence on the growth and distribution of *Pennisetum alopecuroides* (L.) [35].

In this study, the high suitable areas accounts for 60.05% of the total area of China from 87.6–126.7 N and 19.5 to 46.6 E. This trend may be related to the fact that *Pennisetum alopecuroides* (L.) can grow by asexual reproduction. Under all future climate scenarios, the suitable area will expand northward, especially to the northeast, and will change from the unsuitable area to the suitable area from the south of Hainan to Inner Mongolia. At the same time, the north section of the western Plateau and Changbei Mountain area also become more suitable with the trend of climate warming and carbon emission increasing.

Therefore, in the development and utilization of *Pennisetum alopecuroides* (L.) resources under climate change, a more efficient management model should be adopted for the high-suitable expansion areas to promote the development of the resources and undertake the important task of the main output of the resources. However, for areas with low adaptability and degeneration of adaptability, such as tropical areas in China, the degree of development of *Pennisetum alopecuroides* (L.) resources should be controlled to ensure ecological benefits first. The distribution of *Pennisetum alopecuroides* (L.) should be controlled as far as possible without further shrinking under the negative effects of climate change, while noting the possibility of developing *Pennisetum alopecuroides* (L.) plantations in typical newly added suitable areas such as southern Tibet. It should be controlled to make rational overall utilization of *Pennisetum alopecuroides* (L.) resources in different areas and to realize the double benefits of Economy and ecology.

This study focused on the influence of climatic factors on the distribution of *Pennisetum alopecuroides* (L.) without other factors such as soil, human activities and so on, which may also have a greater influence on the distribution of species. Because of short of other more suitable data, our model should need to be refined in future. At the same time, other factors such as community dynamics and interspecific relationships are difficult to quantify. Because the actual distribution area of species is much smaller than its potential distribution area, neglecting these factors may lead to overestimation of the potential distribution area. In addition, human activities also affect species distribution of *Pennisetum alopecuroides* (L.). Therefore, the follow-up research will use reliable multi-source data as far as possible to enrich the factor fields involved in the model.

MaxEnt model has the advantage of higher statistical accuracy and better practical application [36]. Future studies may consider using the coupled model for prediction. Furthermore, studies should be comprehensively considered with much more factors, including soil, altitude, terrain and so on, to improve the accuracy of the predictions in future.

## 5. Conclusions

Based on the MaxEnt model, our work found that the main limiting factor of *Pennisetum alopecuroides* (L.) distribution is the precipitation of the warmest quarter, and the overall effect of temperature is stronger than that of precipitation. With the climate change, the suitable area of *Pennisetum alopecuroides* (L.) was expanding along the axis of the subtropical region of China, and the high suitable area was concentrated in the subtropical region. At the end of the 21^st^ century, the suitable retreat would occur from south to north. The results showed that the suitable degree of *Pennisetum alopecuroides* (L.) in China would gradually decrease, and North China Plain and Changbai mountainous areas might become suitable areas for *Pennisetum alopecuroides* (L.) in future.

## Supporting information

S1 Appendix (CSV)
